# The Stability of Glutamatergic Synapses Is Independent of Activity Level, but Predicted by Synapse Size

**DOI:** 10.3389/fncel.2019.00291

**Published:** 2019-06-27

**Authors:** Dylan P. Quinn, Annette Kolar, Sydney A. Harris, Michael Wigerius, James P. Fawcett, Stefan R. Krueger

**Affiliations:** ^1^Department of Physiology and Biophysics, Faculty of Medicine, Dalhousie University, Halifax, NS, Canada; ^2^Department of Pharmacology, Faculty of Medicine, Dalhousie University, Halifax, NS, Canada

**Keywords:** synaptic plasticity, neurotransmitter release, DREADD, channelrhodopsin, hippocampus, presynaptic specializations, dendritic spines

## Abstract

Neuronal activity is thought to drive the remodeling of circuits in the mammalian cerebral cortex. However, its precise function in the underlying formation and elimination of glutamatergic synapses has remained controversial. To clarify the role of activity in synapse turnover, we have assessed the effects of inhibition of glutamate release from a sparse subset of cultured hippocampal neurons on synapse turnover. Sustained chemogenetic attenuation of release through presynaptic expression of a designer receptor exclusively activated by designer drugs (DREADD) had no effect on the formation or elimination of glutamatergic synapses. Sparse expression of tetanus neurotoxin light chain (TeNT-LC), a synaptobrevin-cleaving protease that completely abolishes neurotransmitter release, likewise did not lead to changes in the rate of synapse elimination, although it reduced the rate of synapse formation. The stability of active and silenced synapses correlated with measures of synapse size. While not excluding a modulatory role in synapse elimination, our findings show that synaptic activity is neither required for the removal nor the maintenance of glutamatergic synapses between hippocampal neurons. Our results also demonstrate that the stability of glutamatergic synapses scales with their size irrespective of their activity.

## Introduction

The mammalian brain has a much higher organizational complexity than any other biological tissue. The cerebral cortex in the mouse, for instance, contains approximately 10^7^ neurons that are connected by more than 10^10^ synapses (Schüz and Palm, 1989). Interconnections are initially laid out by mechanisms regulating neuronal migration as well as the outgrowth of axons and dendrites. The resulting immature circuits, however, are frequently less precise than their mature counterparts and undergo further remodeling, a process that is strongly influenced by changes in the activity of afferent neurons (Hubel et al., 1977). At cellular level, circuit refinement is thought to correspond to the elimination of established glutamatergic synapses and addition of new ones (Grutzendler et al., 2002; Trachtenberg et al., 2002). Frequently occurring through extension and retraction of dendritic spines, the remodeling of glutamatergic synaptic connections is largely limited to a critical developmental period in some brain regions (Grutzendler et al., 2002), but continues to reshape circuits throughout adult life in others (Attardo et al., 2015).

Despite extensive work in this area, the precise role of neuronal activity in the regulation of synapse remodeling is still ill-defined. Glutamate release may facilitate synapse formation (Engert and Bonhoeffer, 1999; Maletic-Savatic et al., 1999; Richards et al., 2005; Kwon and Sabatini, 2011) or be required for the maintenance of glutamatergic synapses (McKinney et al., 1999; Yasuda et al., 2011). This notion is challenged, however, by findings that dendritic spine densities are essentially unaltered in neurons lacking ionotropic glutamate receptors (Lu et al., 2013) or in organotypic cultures of Munc13-1/2 knockout mice essentially devoid of neurotransmitter release (Sigler et al., 2017). Neuronal activity may also act to promote synapse elimination through either of two proposed mechanisms. In the homosynaptic model of activity-dependent synapse elimination, anticorrelated activity of pre- and post-synaptic neurons leads to long-term depression (LTD) of synaptic transmission and weakening of the synaptic contact, ultimately leading to its elimination (Becker et al., 2008; Wiegert and Oertner, 2013; Wiegert et al., 2018). In the heterosynaptic model, activity-mediated competitive interactions exist between neighboring glutamatergic synapses, such that synaptic activation leading to long-term potentiation (LTP) at some synapses elicits concomitant weakening (Lynch et al., 1977; Abraham and Goddard, 1983; Scanziani et al., 1996; El-Boustani et al., 2018; Jungenitz et al., 2018) and subsequent elimination (Bian et al., 2015; Oh et al., 2015) of nearby non-activated synapses. The mechanism of activity-mediated synapse elimination can potentially be further elucidated by assessing the stability of inactive or rarely utilized synapses in active circuits. At inactive synapses, LTD and LTP cannot be induced, and any effect on synapse stability exerted by these homosynaptic plasticity mechanisms is absent. In contrast, any heterosynaptic mechanisms destabilizing synapses should also affect inactive inputs. To address whether and how activity-mediated elimination mechanisms shape neuronal circuits, we have therefore inhibited neurotransmitter release in sparse subsets of hippocampal neurons in dissociated culture, a preparation in which LTD and LTP can be readily induced (Bi and Poo, 1998), and compared the stability of glutamatergic synapses made by these silenced neurons to those of active neurons. Using the same approach, we have also re-investigated the role of glutamate release in synapse formation.

## Materials and Methods

### Hippocampal Cultures and Transfections

Dissociated neuronal cultures were prepared from E18 embryonal Sprague Dawley rat hippocampi of mixed sex. All experiments on animals were approved and performed in accordance with the guidelines and regulations by the Dalhousie University Committee on Laboratory Animals (UCLA Protocol #17–128). Following dissection, hippocampi were incubated in 0.03% trypsin for 15 min and dissociated using a fire-polished Pasteur pipette (Matz et al., 2010). Neurons were diluted in Neurobasal medium supplemented with B27 (Thermo Fisher, Waltham, MA, United States), 0.5 mM glutamine, 25 μM glutamate, and 5% fetal calf serum (FCS) and added at a density of 3–6 × 10^3^cm^-2^ to 60-mm dishes containing 5, 16-mm coverslips coated with 0.1% (wt/vol) poly-L-lysine (Peptides International). After 4 h, the plating medium was replaced with serum-free Neurobasal supplemented with B-27, which attenuated but not completely prevented glial proliferation. For all experiments, cultures were transfected 10–14 days after plating using a calcium-phosphate precipitation protocol as previously described (Matz et al., 2010).

### cDNA Constructs

Synaptophysin-mCherry (Syph-mCh) was generated by fusing the ORF of synaptophysin in-frame to the 5′ end of mCherry cDNA contained in an expression vector derived from pEGFP-C1 (Clontech) in which the cDNA encoding EGFP was excised as previously described (Quinn et al., 2017). Homer1B was a gift from Dr. Carlo Sala. The Homer1B sequence was PCR-amplified with primers encoding AgeI and BamHI sites and ligated into a BspEI/BamHI-cut pEGFP-C1 to create EGFP-Hmr. A Gi/o-coupled inhibitory designer receptor exclusively activated by designer drugs (DREADD) construct based on the human muscarinic acetylcholine receptor M4 was purchased from Addgene (pAAV-hSyn-DIO-hM4DGi-mCherry, plasmid# 44362, described in Krashes et al., 2011). The DREADD sequence (hM4DGi) was excised with NheI/AgeI and cloned into pEGFP-C1 cut with NheI/BspEI to create pE-hM4DGi. Tetanus neurotoxin light chain (TeNT-LC) was purchased from Addgene (pGEMTEZ-TeTxLC, plasmid #32640, described in Yu et al., 2004). Using PCR, we added AgeI and MfeI restriction sites to N- and C-termini of the TeNT-LC coding sequence. Via AgeI and MfeI, TeNT-LC was then cloned into pEGFP-C1 immediately downstream of a cytomegalovirus (CMV) promoter, which created pE-TeNT-LC. To visualize neurons and axons which express hM4DGi and TeNT-LC, we first generated a CMV-Syph-mCh cassette with N- and C-terminal PciI sites using PCR. The CMV-Syph-mCh cassette was then inserted into pE-hM4DGi and pE-TeNT-LC at a unique PciI site to create pE-hM4DGi-Syph-mCh and pE-TeNT-LC-Syph-mCh.

### Assessment of Morphological Plasticity of Glutamatergic Synapses

To assess synapse turnover in dissociated hippocampal neurons, we fluorescently labeled pre- and post-synaptic specializations in separate populations of neurons and recorded the number of stable, newly formed and eliminated synapses that occurred over 24 h. To label presynaptic sites, hippocampal neurons were transfected at 13 DIV with either 20 μg/dish of Syph-mCh (control groups), 20 μg/dish of TeNT-LC-Syph-mCh, or 40 μg/dish of hM4DGi-Syph-mCh. To label post-synaptic densities and dendritic spines, hippocampal neurons were transfected at 14 DIV with 40 μg/dish of EGFP-Hmr. At 16 DIV, coverslips were placed in a circular imaging chamber and imaged with a Zeiss Observer 2.1 inverted microscope using a Photometrics Coolsnap HQ2 camera and SlideBook 6 imaging software. Experiments were performed at 36 ± 2°C in HBS solution containing (in mM) 110 NaCl, 5.3 KCl, 2 CaCl_2_, 1 MgCl_2_, 20 4-(2-hydroxyethyl)-1-piperazineethanesulfonic acid (HEPES), and 25 D-glucose adjusted to pH 7.30. Before fluorescent imaging, the rotational center of each coverslip was determined, and a 10× DIC image was acquired. Without moving the imaging chamber, immersion oil was applied to the 63× (N.A. 1.4) objective and it was brought into the imaging path. Stacks comprising seven images over a distance of 2.1 μm were acquired of neurons showing Syph-mCh positive axons in contact with EGFP-Hmr positive dendrites. These are referred to Day 0 (D0) images. The *XY* coordinates of each neuron were saved and coverslips were returned to the incubator for 24 h. The following day, the rotational center of each coverslip was revisited, and the chamber was rotated until the live 10× DIC image aligned with the DIC image from the previous day. Saved coordinates were then revisited, and a second image stack was acquired for each experiment (D1 images). Before image acquisition, the experimenter was blinded to the experimental conditions. Image stacks were converted to maximum intensity projection images, background corrected, and D0 and D1 images were manually aligned using EGFP-Hmr images. Axons and dendrites which appeared unhealthy (i.e., showed significant structural changes or were missing from D1 images) were excluded from analysis. Co-localizations of Syph-mCh and EGFP-Hmr that had a center-to-center distance less than or equal to 0.8 μm were considered synaptic. By comparing D0 and D1 images, the number of stable, eliminated, and newly formed synapses was recorded. In a subset of experiments, we quantified the intensities of Syph-mCh and EGFP-Hmr puncta by placing segments on the images and measuring the fluorescent intensities of stable, eliminated and newly formed synapses. Synaptic regions of measurement had a size between 0.32 and 0.64 μm^2^. To compensate for expression variability between neurons, the intensity of EGFP-Hmr/Syph-mCh puncta on each post-synaptic neuron was normalized to the average intensity of all analyzed EGFP-Hmr/Syph-mCh puncta for that neuron. All analysis was performed using IPLab software in a blinded fashion. For Figure 3B, 5D,E, the percentage of formed synapses was calculated by dividing the number of formed synapses by the total number of stable and formed synapses. Similarly, the percentage of eliminated synapses was calculated by dividing the number of eliminated synapses by the total of stable and eliminated synapses.

### Electrophysiology

Whole-cell recordings were performed using hippocampal neurons cultures 14–16 days after plating. Borosilicate micropipettes with a resistance of 3–4 MΩ were filled with intracellular solution (pH 7.4) containing (in mM) potassium gluconate 120, NaCl 5, HEPES 10, EGTA 0.5, MgATP 0.4. The bath solution contained (in mM) NaCl 110, HEPES 10, D-glucose 5, KCl 3, CaCl2 2, MgCl2 1, adjusted to pH 7.4. For chemogenetic silencing, the DREADD agonist 11-(1-Piperazinyl)-5*H*-dibenzo[*b,e*][1,4]diazepine was either added to the culture medium at a concentration of 2–10 μM immediately following transfections for chronic silencing experiments, or, for acute experiments, added at a concentration of 10 μM to the bath solution. 10 μM 6,7-Dinitroquinoxaline-2,3(1H,4H)-dione (DNQX), added to the bath at the end of experiments, was used to confirm the glutamatergic nature of synaptic connections recorded. Neurons were visualized by phase-contrast microscopy using a Nikon TE2000 inverted microscope equipped with a 20×, N.A. 0.45 objective (Nikon) and a Hamamatsu ORCA CCD camera. Recordings were acquired using a Multiclamp 700B amplifier (Axon Instruments, Union City, CA, United States), a Digidata 1320A digitizer and AxoGraph software^1^. Voltage-clamped neurons were held at -70 mV. Signals were filtered at 5 kHz and sampled at 10 kHz. Series resistance was monitored and not compensated, and neither series resistance nor input resistance were significantly different in recordings of neurons from control (*R*_s_ = 13.0 ± 1.1 MΩ, *R*_m_ = 118.8 ± 9.5 MΩ, *n* = 26) and chemogenetically silenced cultures (*R*_s_ = 13.4 ± 1.0 MΩ, *R*_m_ = 132.6 ± 14.1 MΩ, *n* = 28, unpaired Student’s *t*-test). To optogenetically stimulate oCHIEF-transfected neurons, a LED light source (SpectraX, Lumencor, Beaverton, OR, United States) was mounted to the back of the microscope and focused through the objective lens. In a set of preliminary experiments, cyan light (470/24 nm) stimuli of 1 ms duration were delivered at various intensities to select an intensity that resulted in reliable excitation of oCHIEF-transfected neurons to be used throughout the study.

### Statistical Analysis

**FIGURE 1:**
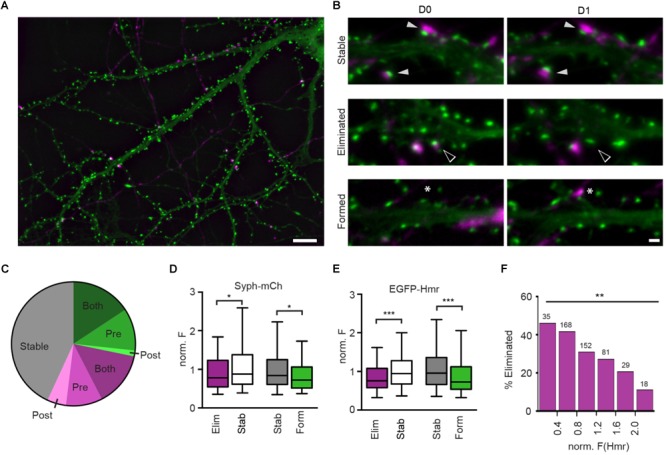
Structural plasticity of synapses in hippocampal culture. **(A)** Overview image of Syph-mCh-expressing presynaptic neurons (magenta) and EGFP-Hmr-expressing postsynaptic neurons (green). Scale bar: 10 μm. **(B)** Micrographs of stable (filled arrowheads), eliminated (open arrowheads) and formed synapses (asterisks). Scale bar: 1 μm. **(C)** Fractions of stable (gray), eliminated (magenta) and formed synapses (green). Shades of green and magenta indicate whether only presynaptic (Pre), post-synaptic (Post) or pre-and post-synaptic compartment (Both) exhibited structural plasticity leading to synapse gain or loss. **(D)** Normalized Syph-mCh fluorescence intensities of eliminated synapses (median 0.78) were significantly lower than those of stable synapses (median 0.88; Mann-Whitney U-test: *U* = 22683, *p* = 0.019). Similarly, newly formed synapses (median 0.72) had less Syph-mCh than stable synapses (median 0.84; *U* = 18243, *p* = 0.012). **(E)** Normalized EGFP-Hmr fluorescence intensities of eliminated synapses were significantly lower than those of stable synapses (Mann-Whitney U-test: *U* = 20788, *p* = 0.0003). Similarly, newly formed synapses had less EGFP-Hmr than stable synapses (*U* = 16396, *p* < 0.0001). For **D,E**, the dataset contained *n* = 320 stable synapses, *n* = 163 eliminated synapses and *n* = 134 formed synapses. **(F)** Binning of data from d shows that synapses with little EGFP-Hmr accumulation and putatively small PSDs are frequently eliminated whereas synapses with large EGFP-Hmr clusters, indicative of large PSDs, are much more likely retained [Chi-square test: *X*^2^(*df* = 5, *n* = 483) = 15.41, *p* = 0.0088]. Numbers above bars show the number synapses analyzed for each bin. ^∗^*p* < 0.05, ^∗∗^*p* < 0.01, ^∗∗∗^*p* < 0.001.

**FIGURE 2:**
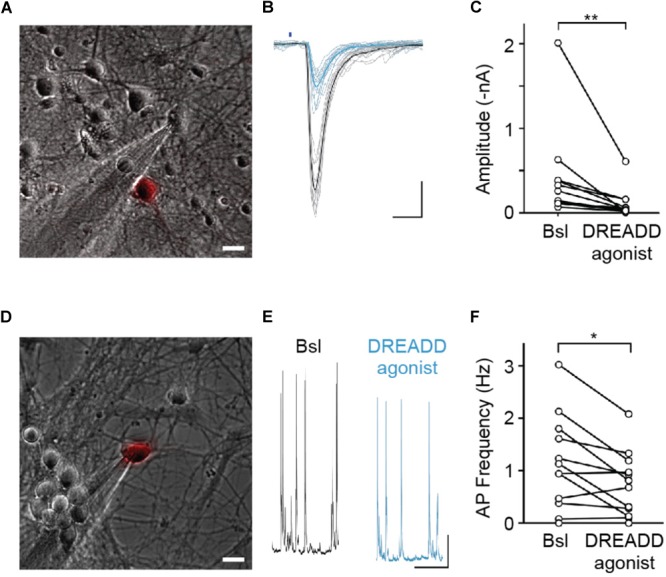
hM4DGi activation leads to a reduction in evoked neurotransmitter release and a decrease in action potential firing frequency. **(A–C)** Neurons transfected with cDNAs encoding hM4DGi, mCherry, and oCHIEF were identified and whole-cell currents were measured in voltage-clamp recordings of adjacent untransfected neurons (scale bar in **A,D**: 10 μm). In neurons in which brief illumination with blue light (denoted by blue bar in **B**) resulted in short-latency EPSCs, DREADD agonist was applied. Black and blue traces depict EPSCs before and after drug application. Scale bars: 10 ms, 500 pA. **(C)** In *n* = 12 experiments, agonist application led to a significant reduction in EPSC amplitude (median amplitudes 200.3 and 39.1 pA before and after drug application, respectively, Wilcoxon Signed Rank Test *Z* = 3.04, *p* = 0.0024). **(D–F)** Neurons were transfected with hM4DGi and mCherry, and whole-cell current clamp recordings were made from transfected neurons to measure the action potential frequency before and after application of DREADD agonist (black and blue traces in **E**). Scale bars: 1 s and 5 mV. **(F)** In *n* = 12 experiments, agonist application led to a significant reduction of AP frequency (median action potential frequencies 1.03 and 0.74 Hz before and after drug application; Wilcoxon Signed Rank Test *Z* = 2.29, *p* = 0.022). ^∗^*p* < 0.05, ^∗∗^*p* < 0.01.

Statistical comparisons were made using GraphPad Prism 6. Data sets were tested for normality with the D’Agostino-Pearson omnibus normality test. Taking the fraction of formed and eliminated synapses onto each neuron as an independent observation, formation and elimination rates over all neurons in a dataset were found to be normally distributed, depicted in graphs as mean ± SEM, and tested with unpaired Student’s t-tests. In contrast, syph-mCh and EGFP-Hmr intensities at individual synapses as well as EPSC amplitudes in electrophysiological experiments were non-normally distributed and depicted in box plots showing median, first and third quartile (box) as well as 5th and 95th percentile (whiskers). To compare fluorescence intensities at individual synapses as well as EPSC amplitudes in chronically silenced (DREADD, TeNT-LC) and control cultures, we used Mann-Whitney U tests. For acute chemogenetic silencing experiments yielding matched samples (Figure 2C,F), we carried out Wilcoxon Signed-Rank tests. The electrophysiological analysis of the fraction of connected neurons shown in Figure 4C involved categorical data and was undertaken using a Chi-squared test. Finally, the analysis of elimination rates of synapses binned for post-synaptic densities (PSD) size (Figure 1F, 5G) was performed using contingency tables and subsequent Chi-squared tests as well as log-linear analysis (Figure 5G). Statistical tests were considered to be significant at a 95% confidence interval, with *p* values reported in the Results section. In figures, asterisks denote significance levels as follows: ^∗^*p* < 0.05, ^∗∗^*p* < 0.01, ^∗∗∗^*p* < 0.001.

## Results

### Synaptic Refinement in Dissociated Rat Hippocampal Neurons

To study the characteristics of synapse refinement in cultures of dissociated hippocampal neurons using time-lapse fluorescent imaging, we transfected neurons at 13 days *in vitro* (DIV) with synaptophysin-mCherry (Syph-mCh), which localizes to synaptic vesicles in presynaptic specializations. At 14 DIV, a separate population of neurons was transfected with EGFP-Homer1B (EGFP-Hmr, Figure 1A,B), a marker for PSDs. At 16 DIV, image stacks were acquired of EGFP-Hmr expressing neurons that co-localized with Syph-mCh expressing axons. On the following day, the same fields were revisited, and a second image stack was acquired. Maximum intensity projections of image stacks were created, and the fractions of stable, eliminated, and newly formed synapses were quantified. On average, 43.1 ± 5.6% (mean ± SEM, 55 neurons) of analyzed synapses remained stable, 28.6 ± 2.3% of synapses were eliminated, and 28.2 ± 3.7% of synapses were newly formed over a 24-hour period (Figure 1C). The structural events that underlie synapse formation and synapse elimination are currently not well understood. Fluorescently labeled pre- and post-synaptic neurons allowed us to classify synapse eliminations and formations according to structural changes occurring in pre- and/or post-synaptic compartments (Figure 1C). Synapse formations predominately involved the simultaneous appearance of pre- and post-synaptic elements in the image on the second day (56% of synapse formations). In a minority of instances, synapse formation occurred by the appearance of a presynaptic bouton to contact a pre-existing dendritic spine (39%) or the extension of a spine to contact a pre-existing presynaptic bouton (5%). For synapse eliminations, most instances involved the simultaneous disappearance of pre- and post-synaptic elements on the second day (50% of synapse eliminations). The remaining synapse eliminations either occurred due to the mobilization of a presynaptic bouton away from a remaining dendritic spine (33%) or the retraction of a dendritic spine from a stable presynaptic bouton (17%). This analysis shows that in cultures of dissociated hippocampal neurons, a significant fraction of synapses is replaced in the course of 24 h and that both pre- and post-synaptic elements contribute to synapse assembly and disassembly.

### Synapse Size Predicts Synapse Survival

Previous studies have suggested a relationship between spine size and synapse stability (Holtmaat et al., 2005; Bastrikova et al., 2008; Pfeiffer et al., 2018). Spine volume scales with parameters such as PSD size and the number of synaptic vesicles in the apposed presynaptic varicosity (Harris and Stevens, 1989; Arellano et al., 2007; Meyer et al., 2014), and it could be hypothesized that synapse size is predictive of its stability. To address this possibility, we identified stable, eliminated, and newly formed synapses on maximum intensity projections of image stacks obtained 24 h apart and quantified the fluorescent intensities of Syph-mCh and EGFP-Hmr puncta, which served as proxy measurements for total synaptic vesicle pool and PSD size, respectively. Intensity measurements for co-localized Syph-mCh and EGFP-Hmr puncta were normalized to the average intensity of all analyzed synapses for each pair of Syph-mCh and EGFP-Hmr expressing neurons. To test if puncta intensities were predictive of synapse stability, we compared the Day 0 Hmr and syph intensities of stable and subsequently eliminated synapses. We found that Syph-mCh and EGFP-Hmr puncta at eliminated synapses were significantly less intense compared to synapses that remained stable (Figure 1D,E). Similarly, a comparison of Day 1 Hmr and Syph intensities of persistent and newly formed synapses revealed that nascent synapses were also significantly less intense compared to stable synapses (Figure 1D,E). The relationship between synapse size and stability became even more apparent when we binned synapses according to Hmr-EGFP fluorescence intensity. This analysis revealed an approximately 4-fold difference in the rate of elimination between synapses with small and large PSDs (Figure 1F). Taken together, these results indicate that synapse size is predictive of synapse stability.

### Synapse Turnover and Function Is Not Affected by Chemogenetic Silencing

Neuronal activity is thought to modulate synapse refinement. However, it has remained unclear if activity is required for synapse turnover and whether activity-dependent modulation of synapse elimination is due to a homosynaptic LTD-like mechanism (Wiegert and Oertner, 2013) or is heterosynaptic, involving local competition between neighboring synapses (Oh et al., 2015). To address these questions, we sought to selectively silence sparse subsets of synaptic afferents in our cultures. For this purpose, we utilized hM4DGi (Armbruster et al., 2007), a G_i_ protein-coupled DREADD activated by the synthetic ligand 11-(1-Piperazinyl)-5*H*-dibenzo[*b,e*][1,4]diazepine (DREADD agonist 21). In an initial set of experiments, we confirmed that expression of hM4DGi in hippocampal neurons allows for attenuation of synaptic transmission through application of DREADD agonist. Hippocampal neurons were sparsely transfected with cDNAs encoding the DREADD hM4DGi, mCherry, as well as the channelrhodopsin variant oCHIEF (Lin et al., 2009), which allowed us to selectively stimulate transfected neurons with brief light pulses. Two days following transfection, we identified transfected neurons, established whole-cell voltage clamp recordings from nearby untransfected neurons, and optogenetically stimulated neurons expressing oCHIEF (Figure 2A). In a subset of recorded neurons in which this procedure reliably elicited EPSCs with short latency, DREADD agonist 21 was added to the extracellular solution (Figure 2B). Acute application of agonist resulted in a 74% reduction of EPSC amplitude (Figure 2C). This result confirms earlier observations (Stachniak et al., 2014) demonstrating that hM4DGi signaling sharply reduces neurotransmitter release at glutamatergic synapses. In a subset of experiments in which cultures had previously been treated for 48–72 h with DREADD agonist 21 before washout of drug and electrophysiological recordings, a second, acute application of the DREADD agonist resulted in a 68% reduction of EPSC amplitude (*n* = 5; data not shown), suggesting little or no desensitization of DREADD signaling during chronic applications of the agonist. In addition to assessing the effect of DREADD signaling on synaptic transmission, we also assessed its effect on the excitability of cultured hippocampal neurons. For this purpose, we transfected neurons with cDNAs encoding hM4DGi as well as mCherry and directly recorded from transfected neurons in current clamp to determine the rate of spontaneously occurring action potentials before and after application of DREADD agonist 21 (Figure 2D–F). Acute activation of hM4DGi resulted in a significant reduction of the action potential frequency of 36% on average (Figure 2F). Taken together, our results show that hM4DGi can be employed to strongly reduce synaptic activity in subsets of synapses in chronic experiments. They also demonstrate that hippocampal neurons in our cultures exhibit considerable spontaneous activity and are well suited to study the modulation of synapse turnover by neuronal activity.

**FIGURE 3:**
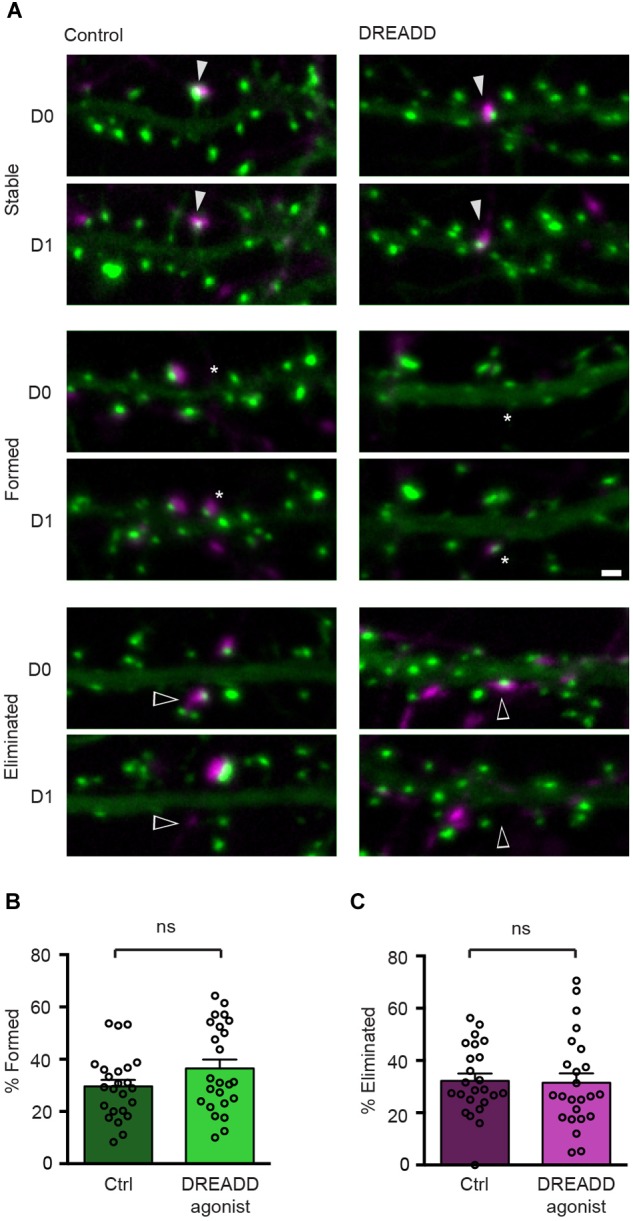
DREADD-mediated silencing does not affect synapse turnover. **(A)** Micrographs of stable (filled arrowheads), eliminated (open arrowheads) and formed (asterisks) synapses in control and DREADD-treated cultures. **(B)** The fraction of newly formed synapses in DREADD-treated cultures (36.5 ± 3.4%) and control cultures (29.6 ± 2.5%) was not significantly different (*n* = 24 neurons over three cultures; *p* = 0.111, *t*(46) = 1.62, Student’s *t*-test). **(C)** The fraction of eliminated synapses in DREADD-treated cultures (31.5 ± 3.6%) and control cultures (32.2 ± 2.7%) was similar (*n* = 24; *p* = 0.865, *t*(46) = 0.17, Student’s *t*-test). Data are shown as mean ± SEM. Scale bar = 1 μm.

To determine the effect of chemogenetic silencing of neurons on the stability of their efferent synapses, we expressed hM4DGi along with Syph-mCh in a small subset of neurons and labeled glutamatergic post-synaptic specializations in a second subset of neurons in a subsequent transfection with a plasmid encoding EGFP-Hmr. Immediately following the second transfection, DREADD agonist 21 was added to the culture medium of half of the transfected cultures, and the remaining untreated cultures served as controls. After 2 days of chemogenetic silencing, synapses between Syph-mCh and hM4DGi-expressing presynaptic neurons and post-synaptic neurons expressing EGFP-Hmr were identified in maximum intensity projections of acquired image stacks (D0 in Figure 3A). The same neurons were then re-imaged 24 h later to identify stable, eliminated and newly formed synapses (D1 in Figure 3A). Analysis of these experiments revealed that synapse formation was unaffected by chemogenetic silencing (Figure 3B). Similarly, chemogenetic silencing did not affect the rate of elimination of synapses with strongly reduced activity (Figure 3C). These results suggest that the strong attenuation of glutamate release in chemogenetically silenced neurons neither affects the formation nor the elimination of efferent synapses.

**FIGURE 4:**
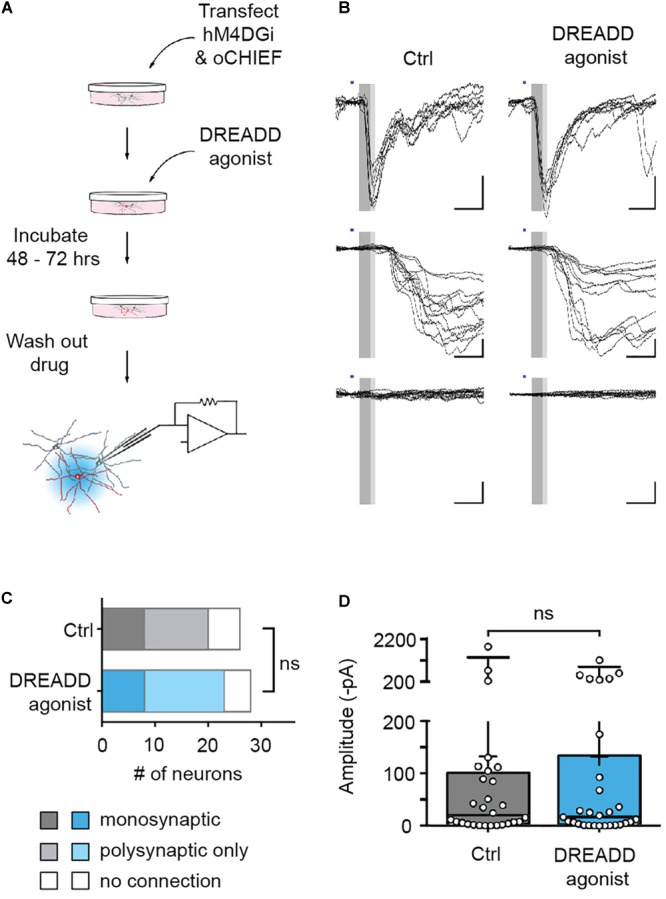
Efferent synapses of chemogenetically silenced neurons are functionally unaltered. **(A)** Experimental design. **(B)** Recordings from cultures chronically treated with DREADD agonist (right) and cultures to which no agonist was applied (control, left). Illumination with blue light (1 ms duration, blue bars) either evoked synaptic currents with short latency (<6.5 ms, dark gray area in sample traces), and low jitter (putative monosynaptic connections, top row), synaptic currents with longer latencies (>6.5 ms) and larger jitter (putative polysynaptic, i.e., indirect connections, middle row), or no responses at all (bottom row). **(C)** In recordings from 26 untreated and 28 agonist-treated cultures, there was no significant difference in the fraction of monosynaptic, polysynaptic or unconnected neurons between control and DREADD agonist-treated cultures [Chi square test: *X*^2^(2, *n* = 54) = 0.35, *p* = 0.84]. Monosynaptic GABAergic connections (two in control cultures, one in DREADD agonist-treated cultures) were excluded from the analysis. **(D)** Post-synaptic current amplitudes observed within 8 ms after stimulus onset (complete gray area in **B**) in control cultures (median 13.7 pA) and cultures treated with DREADD agonist (median 14.4 pA) were not significantly different (Mann Whitney U rank sum test: *U* = 361.5, *p* = 0.97). Medians of the distributions are indicated by large black bars, means by small black bars, first and third quartiles by box boundaries and 5th and 95th percentiles by whiskers. Circles represent recordings from individual neurons.

The reversibility of DREADD-mediated silencing allowed us to complement these morphological findings with an assessment of the functional consequences of chronic hM4DGi activation. To this end, we co-transfected neurons with plasmids encoding hM4DGi, mCherry, and oCHIEF at 14 DIV and added DREADD agonist to the media to half of the cultures immediately after transfection. Two to three days after transfection, the DREADD agonist was removed, transfected neurons were identified (9.1 ± 0.8% of all neurons in eight independent transfections; data not shown) and whole-cell voltage-clamp recordings from nearby untransfected neurons were established (Figure 4A). Subsequent light-evoked stimulation resulted in several different outcomes. In a subset of recordings, optogenetics stimulation elicited post-synaptic currents with short latencies (less than 6.5 ms following stimulation onset). These responses likely arose from monosynaptic, i.e., direct synaptic connections from a transfected neuron onto the recorded neuron (Figure 4B, top row). In another subset of recordings, optogenetics stimulation led to post-synaptic currents with larger latency, likely resulting from polysynaptic, i.e., indirect, connections (Figure 4B, middle row). Finally, in some neurons, no post-synaptic currents could be elicited with optogenetics stimulation. In recordings from a total of 54 neurons, we found no significant differences in the fraction of monosynaptic and polysynaptic connections as well as unconnected neurons (Figure 4C). Moreover, post-synaptic current amplitudes observed within 8 ms after stimulus onset, likely arising from monosynaptic connections, were not significantly different in control cultures and cultures treated with DREADD agonist (Figure 4D). Taken together, morphological and electrophysiological experiments employing hM4DGi suggest that chemogenetically silenced presynaptic neurons form and maintain synapses to a similar degree as their active counterparts.

### Blockade of Neurotransmitter Release by Expression of TeNT-LC Reduces the Rate of Synapse Formation but Has No Effect on Synapse Elimination

**FIGURE 5:**
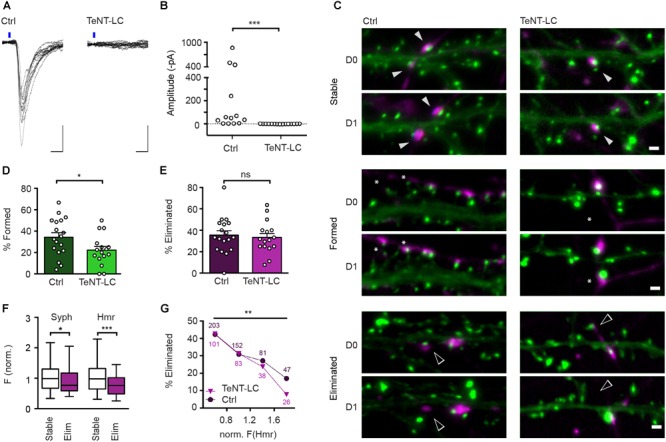
TeNT-LC expression has no effect on synapse elimination but significantly reduces formation. **(A)** Neurons were transfected with a plasmid encoding oCHIEF and either a plasmid with cDNAs encoding TeNT-LC and Syph-mCh, or a plasmid only encoding Syph-mCh (control). Two days following transfection, transfected neurons were identified and nearby untransfected neurons were recorded in whole-cell voltage clamp. Shown are responses to optogenetic stimulation in neurons from controland TeNT-LC-transfected cultures. Scale bars: 10 ms, 100 pA. **(B)** In recordings from 15 neurons in TeNT-LC-transfected cultures, optogenetics stimulation failed to induce any responses. In contrast, optogenetics stimulation elicited reliable post-synaptic currents in the majority of 13 control cultures (Mann Whitney U test: *U* = 184.5; *p* < 0.001). **(C)** Example micrographs of stable (filled arrowheads), eliminated (open arrowheads) and formed synapses (asterisks) in control and TeNT-expressing groups. Scale bar = 1 μm. **(D)** The fraction of synapses formed by neurons expressing TeNT-LC (22.1 ± 3.5%, *n* = 16) was significantly lower than that of control neurons (34.3 ± 4.2%, *n* = 18; unpaired Student’s *t*-test: *t*(32) = 2.19, *p* = 0.036). **(E)** The fraction of eliminated synapses made by neurons expressing TeNT-LC (33.3 ± 3.8%) was indistinguishable from the that of control neurons 35.5 ± 4.0; *t*(32) = 0.39, *p* = 0.696, Student’s *t*-test). Data are shown as mean ± SEM. **(F)** Comparison of Syph-mCh and EGFP-Hmr fluorescence intensities at stable (*n* = 168, white box plots) and eliminated synapses (*n* = 80, purple box plots) from TeNT-LC expressing neurons. Eliminated synapses had lower normalized fluorescence intensities for Syph-mCh and EGFP-Hmr (median of 0.77 and 0.76, respectively) than their stable counterparts (0.99 and 0.98). These differences were significant for Syph-mCh (Mann–Whitney U test: *U* = 5577, *p* = 0.0303) and EGFP-Hmr (*U* = 4784, *p* = 0.0002). **(G)** Synapses were binned according to EGFP-Hmr fluorescence as a measure of synapse size and the fraction of eliminated synapses determined in each size bin. The fraction of eliminated synapses made by TeNT-LC-expressing neurons decreased with increased synapse size [Chi square test: *X*^2^(3, *n* = 248) = 13.4, *p* = 0.0038]. This decrease paralleled the decrease of the elimination rate seen in synapses from control neurons re-binned dataset from Figure 1; *X*^2^(3, *n* = 483) = 14.47, *p* = 0.0021). There was no significant difference in the rate of elimination between control and TeNT-LC-silenced synapses (Log-linear analysis: *G*^2^(4, *n* = 731) = 1.50, *p* = 0.83). Data labels indicate numbers of synapses contained in each bin for TeNT-LC-silenced synapses (triangles) and Ctrl synapses (circles). ^∗^*p* < 0.05, ^∗∗^*p* < 0.01, ^∗∗∗^*p* < 0.001.

Chemogenetic silencing is readily reversible and allowed us to functionally assess its effects on the stability of synaptic connections in electrophysiological recordings. However, DREADD-mediated inhibition does not completely abolish synaptic transmission, and it can be argued that the residual activity might be fully sufficient to drive the activity-induced elimination of these synapses. To address this possibility, we sought to employ a complementary approach that completely, though irreversibly, prevents transmission at glutamatergic synapses. TeNT-LC, a protease that specifically cleaves the R-SNARE synaptobrevin, blocks evoked neurotransmitter release (Schiavo et al., 1992; Sando et al., 2017). Sparse expression of TeNT-LC in approximately 10% of all neurons did not significantly affect the neuronal activity in the cultures overall as evidenced by unchanged frequency (TeNT-LC-expressing cultures: 9.9 Hz, control: 10.9 Hz; Mann Whitney U test: *U* = 125.5, *z* = 0.08) and mean amplitude (TeNT-LC-expressing cultures: 34.8 pA, control: 33.8 pA; Mann Whitney U test: *U* = 131.5, *z* = -0.11) of spontaneous post-synaptic currents (data not shown). However, consistent with previous studies, neurotransmitter release was completely abolished from TeNT-LC expressing neurons, as demonstrated by the absence of light-evoked EPSCs from hippocampal neurons co-transfected with TeNT-LC and oCHIEF (Figure 5A,B). We then assessed the effect of this activity blockade at a sparse subset of synapses on synapse turnover. Cultures were sequentially transfected with TeNT-LC/Syph-mCh or Syph-mCh alone at 13 DIV and EGFP-Hmr at 14 DIV and synapses were imaged over 24 h (Figure 5C). Silencing of neurotransmission via TeNT-LC expression did not affect synapse elimination (Figure 5E). This finding demonstrates that abolishing neurotransmission at individual synapses has no net effect on their stability and confirms the results obtained with DREADD-mediated silencing. Interestingly, however, synapse formation was significantly reduced by TeNT-LC expression compared to control cultures (Figure 5D).

### The Relationship Between Size and Stability Is Maintained at Silent Synapses

Initial experiments outlined in Figure 1 suggest that the size of glutamatergic synapses is predictive of their stability such that smaller synapses are more likely to be eliminated than larger synapses. Previous studies have suggested that presynaptic neurotransmitter release probability (Matz et al., 2010; Holderith et al., 2012) as well as the abundance of post-synaptic glutamate receptors (Nusser et al., 1998; Takumi et al., 1999) scale with synapse size, and it is conceivable that these alterations in the strength of synaptic transmission are causal to observed changes in synapse stability. To address this possibility, we re-assessed the relationship between measures of synapse size and turnover at synapses silenced by presynaptic expression of TeNT-LC. Silenced synapses that were eliminated in the course of the experiment on average contained less Syph-mCh and EGFP-Hmr than silenced synapses that remained stable during the observation period (Figure 5F). Moreover, when we binned TeNT-LC-silenced synapses for Hmr-EGFP fluorescence as a measure of PSD size, the smallest synapses were more than five times as likely to be eliminated than the largest synapses in the distribution (Figure 5G). Finally, when we compared the size/stability relationships of silenced synapses with that of active control synapses, we found no significant difference. Together, these results indicate that activity-independent mechanisms, rather than increases in the reliability and efficacy of synaptic transmission, are responsible for the increased stability of large synapses.

## Discussion

In this study, we have characterized the formation and elimination of glutamatergic synapses using time-lapse imaging of hippocampal neurons in which pre- and post-synaptic structures were visualized with fluorescently tagged proteins. This methodology, which differs from the conventionally taken approach to assess spine plasticity as a proxy for the turnover of glutamatergic synapses, was necessary in order to address the consequences of presynaptic manipulations on synapse stability. In the initial analysis, we observed a surprisingly high degree of synapse elimination and formation. In part, the level of synapse turnover may be due to the preparation used. Interestingly, a recent *in vivo* study of spine dynamics in CA1 pyramidal neurons in the adult rodent hippocampus has reported rates of spine eliminations in the order of 40% over an observation period of 4 days (Pfeiffer et al., 2018). This degree of spine turnover far exceeds the morphological plasticity found in many regions of the neocortex and is likely due to intrinsic properties of hippocampal neurons (Attardo et al., 2015; Pfeiffer et al., 2018). In addition, some of the discrepancy in the rate of synapse turnover between our and earlier studies may be owed to differences in methodology. We find that approximately a third of all synapse formations and eliminations involve the mobilization of presynaptic specializations at stable dendritic spines, which would have not been detected in studies focusing on spine dynamics. In light of these considerations, the rate of synapse turnover seen in our *in vitro* preparation appears to roughly reflect the degree of morphological synaptic plasticity occurring in the hippocampus *in vivo*.

Most saliently, our results demonstrate that synaptic transmission is not necessary for either the retention or the elimination of glutamatergic synapses, although it remains possible that activity has a modulatory role in synapse turnover. Moreover, the finding that glutamatergic synapses between hippocampal neurons display uniform rates of elimination irrespective of their overall activity level places important constraints on mechanistic models of how synaptic activity influences synapse stabilization and elimination. Previous studies have suggested that the elimination of glutamatergic synapses is a consequence of either homosynaptic LTD (Becker et al., 2008; Wiegert and Oertner, 2013) or, alternatively, heterosynaptic competition between neighboring contacts (Oh et al., 2015). Acting in isolation, a homosynaptic activity-dependent mechanism of synapse weakening should spare inactive synapses, making them less prone to synapse elimination. Conversely, any heterosynaptic, competition-based mechanism should place inactive synapses at a disadvantage, leading to increased elimination of synapses with attenuated or completely inhibited synaptic transmission. Interestingly, our findings demonstrate that inactive glutamatergic synapses are neither preferentially maintained nor eliminated, suggesting that any modulation of synapse turnover by neuronal activity is highly balanced and likely involves multiple mechanisms of activity-dependent synaptic weakening and/or strengthening. In a model consistent with our findings, LTD-mediated homosynaptic weakening of synapses may be counteracted by LTP-elicited strengthening (Wiegert et al., 2018). In a population of silenced or relatively inactive synaptic contacts, lack of LTP as well as LTD induction would leave the rate of elimination unchanged. In an alternative, not mutually exclusive model, homosynaptic and heterosynaptic mechanisms of synapse elimination may coexist. In this scenario, a relative lack of LTD-mediated synapse weakening at silent synapses may be compensated by increased weakening due to heterosynaptic mechanisms, leading to overall unchanged synapse stability. We propose that such a finely tuned balance between LTP- and LTD-mediated influences on synapse stability, or between homosynaptic and heterosynaptic processes of synapse elimination, ensures the long-term survival of appropriate, yet little utilized synaptic connections in neuronal circuits.

While the stability of glutamatergic synapses is unaffected by their activity level, we found that it correlates strongly with measures of synapse size. This result is reminiscent of findings in previous studies showing that the permanence of dendritic spines is predicted by spine head volume (Holtmaat et al., 2005; Bastrikova et al., 2008; Pfeiffer et al., 2018), which scales with PSD size (Harris and Stevens, 1989; Arellano et al., 2007; Meyer et al., 2014). Large spines with extensive PSDs usually possess high numbers of AMPA receptors on their post-synaptic surface (Nusser et al., 1998; Matsuzaki et al., 2001), and it could be hypothesized that the increased stability of large synapses is owed to efficacious synaptic transmission. Our study demonstrates, however, that the relationship between size and stability is preserved in synapses silenced by presynaptic expression of TeNT-LC. This finding argues that the cause for the increased stability of large glutamatergic synapses is unrelated to their transmission properties and may rather be more structural in nature. It is interesting to note that, apart from the effect on the recruitment and dispersion of post-synaptic AMPA receptors, induction of LTP and LTD have also been shown to strongly affect F-actin turnover in spines and thus alter spine head volume (Matsuzaki et al., 2004; Okamoto et al., 2004; Zhou et al., 2004). The structural effects of F-actin accumulation and disassembly as well as the resulting changes in the amount of scaffolding and cell adhesion proteins may affect synapse stability independent of any changes in synaptic transmission. Our work shows that large synapses generated in this way can be inactive for long periods of time yet maintain their stability.

In contrast to the lack of an overt effect of silencing on the synapse elimination, blockade of neurotransmitter release through expression of TeNT-LC led to a significant attenuation of synapse formation. This finding is consistent with the view that glutamate release, although not required, facilitates synapse formation (Engert and Bonhoeffer, 1999; Maletic-Savatic et al., 1999; Richards et al., 2005; Kwon and Sabatini, 2011; Sigler et al., 2017). Our findings replicate observations in the retina showing that expression of TeNT-LC in a subset of bipolar cells leads to a reduction in the number of synapses formed by silenced neurons onto retinal ganglion cells (Kerschensteiner et al., 2009). They are also in agreement with a study reporting lower spine densities in CA1 pyramidal neurons of mice expressing TeNT-LC in the forebrain (Sando et al., 2017). We have extended these findings by demonstrating that a milder attenuation of evoked neurotransmitter release by DREADD-mediated inhibition does not lead to a similar effect on synapse formation. Taken together, these findings are consistent with the notion that the formation of glutamatergic synapses is indeed facilitated by a form or degree of glutamate release that is inhibited by TeNT-LC, but unaffected by DREADD-activated G_i_ protein signaling. However, it cannot be excluded that TeNT-LC-mediated cleavage of synaptobrevin exerts its effect on synapse formation through mechanisms other than preventing neurotransmitter release (Sabo and McAllister, 2003; Tojima et al., 2007). Additional studies are clearly required to resolve the controversy surrounding the role of glutamate release in the formation of glutamatergic synapses.

## Data Availability

The datasets generated for this study are available on request to the corresponding author.

## Ethics Statement

All experiments on animals were approved and performed in accordance with the guidelines and regulations by the Dalhousie University Committee on Laboratory Animals (UCLA Protocol #17–128).

## Author Contributions

SK and DQ conceived and designed the experiments and wrote the manuscript. DQ, SH, MW, and JF performed and analyzed the morphological experiments. SK performed and analyzed the electrophysiological experiments. AK established the neuronal cell cultures and contributed plasmids.

## Conflict of Interest Statement

The authors declare that the research was conducted in the absence of any commercial or financial relationships that could be construed as a potential conflict of interest.

## Footnotes

1 http://www.axograph.com
